# Clinical Effects of Pulmonary Rehabilitation in Very Old Patients with COPD

**DOI:** 10.3390/jcm12072513

**Published:** 2023-03-27

**Authors:** Marc Spielmanns, Sofia-Theresia Schulze, Erhan Guenes, Katarzyna Pekacka-Falkowska, Wolfram Windisch, Anna Maria Pekacka-Egli

**Affiliations:** 1 Pulmonary Medicine, Zuercher RehaZentren, Klinik Wald, 8636 Wald, Switzerland; 2 Department for Pulmonary Medicine, Faculty of Health, University Witten Herdecke, 58455 Witten, Germany; windischw@kliniken-koeln.de; 3 Department for History and Philosophy of Medicine, Poznan University of Medical Sciences, 61701 Poznan, Poland; 4 Department of Pneumology, Cologne Merheim Hospital, Kliniken der Stadt Koeln, 51109 Koeln, Germany; 5 Department of Neurorehabilitation, Zuercher RehaZentren, Klinik Lengg, 8008 Zurich, Switzerland

**Keywords:** pulmonary rehabilitation, elderly patients, FIM, 6-Minute Walk Test, feeling thermometer

## Abstract

Background: Pulmonary rehabilitation (PR) improves physical and mental performance as well as quality of life in patients with chronic obstructive pulmonary disease (COPD). However, data on outcomes in very old patients are insufficient. We analyzed whether the elderly with COPD benefit in a similar way to younger patients from participation in an inpatient PR according to the assessments usually collected. Methods: Data from 3173 patients with COPD were retrospectively analyzed. Patients were referred to PR at the Zurich RehaZentren, Switzerland, between January 2013 and December 2019. PR was performed 6 days per week with an average duration of 18.85 days. Functional Independence Measurement (FIM), Feeling Thermometer (FT), and 6-Minute Walk Test (6MWT) were recorded on admission and discharge. Results: In all age groups, the 6MWT and FT improved significantly. FIM results also showed a significant increase. The results of the different age groups showed no significant differences in percentage improvements according to the assessments that were considered. Conclusions: All patient groups with COPD, even the oldest (>85 years), benefited from PR regardless of their age and according to the assessments. Prospective studies are needed to support this hypothesis.

## 1. Introduction

Pulmonary rehabilitation (PR) has been shown to improve dyspnea, fatigue, exercise capacity and quality of life (QoL) in patients with chronic obstructive pulmonary disease (COPD) or other pulmonary conditions [1,2,3]. PR is defined as a “comprehensive intervention based on a thorough patient assessment, followed by patient-tailored therapies which include, but are not limited to, exercise training, education, and behavior change, designed to improve the physical and psychological condition of people with chronic respiratory disease and to promote the long-term adherence of health-enhancing behaviors” [4].

Generally, patients suffering from COPD benefit from participation in a PR, and the referral criteria are well defined. However, most existing data according to results of PR represent a younger population, and the question arises to what extent these data also represent older people. Age-associated changes in the integrative physiology of exercise, including declining lung function, play a role in promoting multimorbidity in the elderly through limitation in physical function. It is well known that age affects the body in very basic ways, so “tailored therapy” for younger people may not suit and be successful in an older population.

As a matter of fact, the elderly is increasing in number and age [5]. COPD, together with other chronic lung diseases, are the third leading cause of death in people aged 65 years and older. Lowery et al. pointed out that pulmonary diseases have significant consequences for the aging population [6]. Since the proportion of older people in the total population is constantly increasing, the question arises as to whether the investment in PR in older patients is justified. Thus, the benefit of PR for the elderly is rather questionable. To depict the rapidly aging population accurately, it seems to be important to perform studies exclusively analyzing this patient group.

To summarize, there is a need for more evidence which relate to the very old population with COPD (>80 years) and to what extent they might benefit from PR.

We aimed to determine whether older people with COPD would show similar benefits to younger people, following the completion of an inpatient PR program. We hypothesized that PR in very elderly patients with COPD is as effective as in the younger population.

## 2. Materials and Methods

### 2.1. Design

We retrospectively analyzed the records of 3173 patients with an ICD-10-CM Code: J44.00–J44.99 diagnosis who had been referred for inpatient PR to the Zürcher RehaZentren, Klinik Wald, Switzerland between January 2013 and December 2019. Only patients with COPD II–IV according to the current guideline were included in the study. COPD was defined as a heterogeneous lung condition characterized by chronic respiratory symptoms (dyspnea, cough, sputum production and/or exacerbations) due to abnormalities of the airways (bronchitis, bronchiolitis) and/or alveoli (emphysema) that cause persistent, often progressive, airflow obstruction. All participants were on specific medication adjusted to the current guidelines. If not, the medication was extended accordingly.

**Figure 1:**
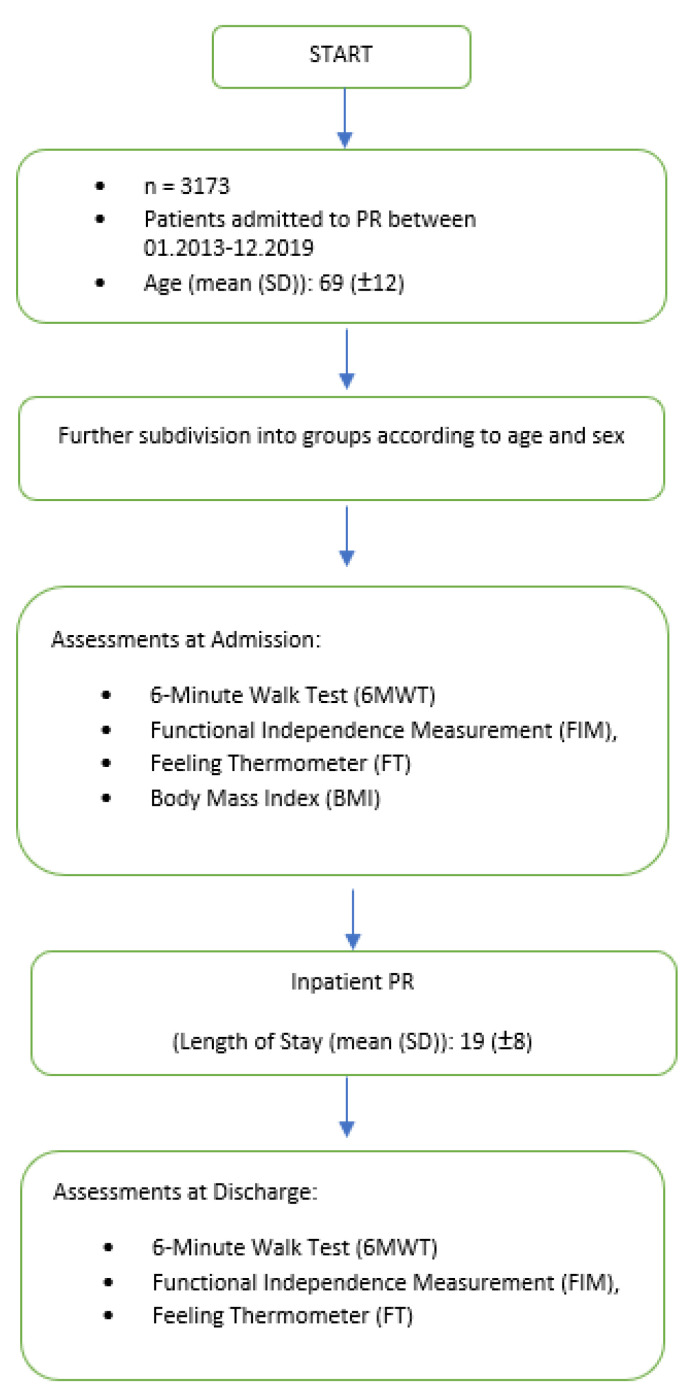
Study criteria and design.

The length of stay was approximately 18.85 days (SD ± 8.17) on average, and PR was performed 6 days per week and included upper and lower extremity muscle strengthening exercises, cycling, treadmill training, and respiratory training. We used the German version of the program RehaTIS^TM^ by Softsolution, International AG, 15830 Lahti, Finland to record and control the individual rehabilitation process of each participant, including all therapies and procedures. During inpatient PR, several pieces of data were collected and documented in the Clinic’s information system, PHOENIX^TM^ (Phoenix^TM^, CompuGroup Medical AG, Bern, Switzerland). Pulmonary function testing (PFT) was not collected from all patients, which is why these data were not included in the evaluation for several reasons (e.g., recent exacerbation, PFT was already performed prior to the PR, oxygen therapy, patient refused PFT). Results of the Functional Independence Measurement (FIM), Feeling Thermometer (FT), and 6-Minute Walk Test (6MWT) were recorded on admission and discharge, as well as the body mass index (BMI) on admission. The collected data of the whole cohort were divided into groups according to age (<60, 61–70, 71–80, 81–85, >85 years) and sex. All patients gave written informed consent. The Ethics Committee of the University of Witten-Herdecke, Germany approved the study protocol (No 110-2021). The study design and criteria are shown in Figure 1.

### 2.2. PR Intervention

The patients participated in a multimodal inpatient PR of 19 days on average. Each patient exercised according to a protocol especially written for the individual severity of the disease. It involved individualized exercise training, including aerobic exercise and strength training. The intensity of the monitored endurance training sessions was adjusted continuously, with the aim of achieving the maximum tolerated exercise load during each training session [7]. When a drop in oxygen saturation was observed, oxygen was offered to maintain the oxygen saturation at >90%.

The PR program presented here was not specifically designed for elderly patients, but was much more disease oriented, especially for patients with COPD. Respiratory physiotherapy consisted of teaching breath control (pursed lip breathing, secretion mobilization, and diaphragm breathing), energy-saving techniques, and controlled coughing exercises along with the activating physiotherapy. Twice a week (1 h each), all patients participated in educational sessions which, in addition to self-management, included topics such as coping skills, nutritional interventions, self-medication options, management of infections and exacerbations, dyspnea, use of oxygen, as well as activities of daily living. The contents of the educational program were based on the concept “Living well with COPD” by Jean Bourbeau [8]. Topics for elderly patients as part of the training program and educational program were additionally addressed, such as physical activity, general health, frailty, disability, sleep quality, and psychosocial functioning. Nutritional- and diabetes advice was offered for the underweight and overweight patients. As an important therapeutic intervention in severely ill COPD patients, we provided nutritional supplementation in form of a higher calorie intake, particularly for those with malnutrition. To produce less CO_2_, we chose high-fat supplements with a lower respiratory quotient value than high-carbohydrate supplements [9]. All members of the team of this PR program were experienced professionals in the following fields: chest physicians, physiotherapists, respiratory therapists, nurses, psychologists, behavioral specialist, exercise physiologists, nutritionists, occupational therapists, and social workers.

If needed, the patients could join a smoking cessation program organized by the clinics. Psychosocial support was offered if needed.

Only rarely non-invasive ventilation was used during the training sessions in some patients. To participate in this comprehensive inpatient PR, the effect of the chronic respiratory disorder on different aspects of the patient’s health status, for instance: symptoms, physiological performance, quality of daily life, activities, and health care utilization, were measured. Most patients were referred to PR from an acute hospital in the region of the Kanton of Zurich, Switzerland after overcoming acute COPD exacerbations. In the case of a few participants, PR was even prescribed electively in stable condition of the disease.

### 2.3. Exercise Capacity

The exercise capacity was measured once on admission and once on PR discharge with the 6MWT [10]. It was performed according to the guidelines of the American Thoracic Society (ATS) [11]. Due to organizational and personnel issues, the 6MWT could only be performed once on admission and discharge. It is known that the effect of learning on the walking distance could be large enough to be clinically important when the 6MWT is used to evaluate response to treatment or change over time [11]. The test was observed and evaluated by experienced, trained examiners. Recently, a minimally important difference (MID) of 20 m to 30 m was determined for patients surviving acute respiratory failure or acute respiratory distress syndrome [12]. The 6MWT provides information on the patient’s ability to perform daily activities and correlates with formal measures of quality of life (QoL) [13]. Changes in the 6MWD after therapeutic interventions correlate with subjective improvement in dyspnea.

### 2.4. Functional Independence Measure

The FIM evaluated the activities of daily living (ADL) and represents efficiency in daily improvement [14]. Out of 18 items 13 defined motor functions and 5 items defined cognitive functions. FIM change scores associated with MID were 22 for the total FIM, motor FIM, and cognitive FIM, respectively. The FIM explores the severity of an individual’s physical and psychological disability, especially of rehabilitation patients. The FIM uses the level of assistance an individual needs to grade functional status from total independence to total assistance. If performed on admission and discharge, these measures can be used to assess change in patient motor and/or cognitive status [14].

Evaluation of the efficacy of PR in patients with confirmed COPD and respiratory failure using FIM determined the degree of disability experienced by patients and the progress they made during PR [15].

### 2.5. Feeling Thermometer

The Feeling Thermometer (FT) is an instrument which enables therapeutics to assess the health state of a patient, for example before and after treatment [16]. This assessment is applicable for almost every disease [17]. Measurements of the direction of attitude and the degree or intensity of the feeling are visualized. Hereby, the respondents are given an opportunity to make fine-graded distinctions using what is essentially a continuous scale, as with a thermometer. The MID for the FT in patients with chronic airflow limitation is approximately 5 to 8 units on the 0 to 100 scale [18].

### 2.6. Duration of Inpatient PR

The duration of inpatient PR seems an important predictor of outcome, since it could show to what extent participation in a PR program is approximately needed for a patient to receive a clinical benefit. However, other studies have shown mixed results regarding the necessity of a longer stay in hospitals or rehabilitation facilities [19,20,21,22]. Only full days of PR were considered. The days of admission and discharge were also included.

### 2.7. BMI

Body Mass Index (BMI) is a measure for indicating nutritional status in adults [23]. The World Health Organization (WHO) defines it as a person’s weight in kilograms divided by the square of the person’s height in meters (kg/m^2^) [24].

The connection between BMI and the benefit of PR seems at some points contradictory and not fully clear. It appears that the very obese as well as very underweight patients profit largely from PR, whereas normal weighted or slightly obese patients do not gain that benefit [25]. Clinical and functional baseline findings do not predict the response to PR in COPD. However, the greater efficacy in patients with BMI >25 may be due to a greater deconditioning in overweight patients [26]. While the extent of gained benefit differs, all patient groups benefit from PR regardless of their BMI [23].

### 2.8. Statistics

All continuous variables were described using mean and standard deviation (SD). All discrete variables are presented as absolute and relative numbers. Boxplots represent 6MWT, FIM, and the Feeling Thermometer across the different age cohorts. Differences in increase were analyzed with Kruskal–Wallis tests and Mann–Whitney U tests with Bonferroni correction as post hoc tests. A *p*-value of 5% was considered significant. All analysis were conducted using R version 4.2.1.

## 3. Results

Data from 3157 patients (1713 men and 1443 women) participating in PR between January 2013 and December 2019 were analyzed. The baseline characteristics are provided in Table 1.

**Figure 2:**
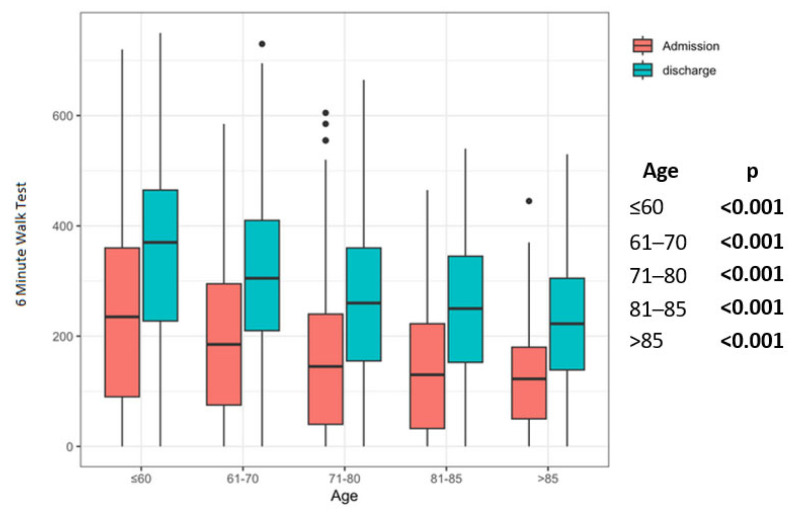
Pre–post comparison results of 6 Minute Walk Test in the age groups. Legend: Statistical significance is marked by bold values.

**Figure 3:**
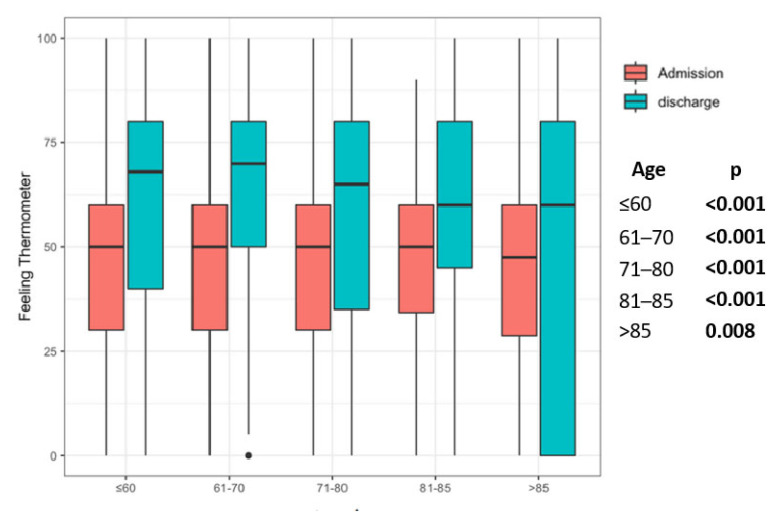
Pre–post comparison results of Feeling Thermometer in the age groups. Legend: Statistical significance is marked by bold values.

**Figure 4:**
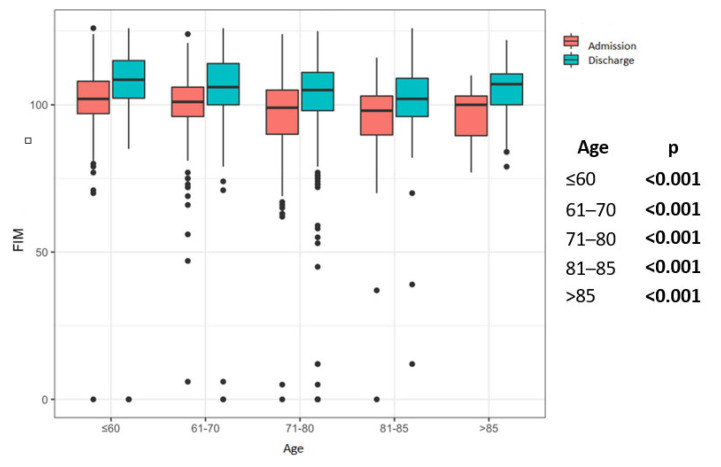
Pre–post comparison results of Functional Independence Measure (FIM) in the age groups. Legend: FIM, Functional Independence Measurement; Statistical significance is marked by bold values.

All patient groups improved significantly in the 6MWT, regardless of the age group (Figure 2). This could also be shown in group comparisons. In all patient groups considered, the FT was significantly better at discharge than on admission (Figure 3). All groups showed a significant increase in FIM at discharge in comparison to admission (Figure 4).

Table 2 provides the percentage improvement in the pre–post comparison with respect to FIM, FT, and 6MWT. It shows that the percentage improvement for the 6MWT and FT between the age groups was significant in favor of the older patient group. The improvements in the FIM score were significant for all age groups but not in the comparison between the age groups. This was also true for the Deltas (absolute values for the pre–post comparison).

## 4. Discussion

In a large cohort, these analyses showed that participation in an inpatient PR program increased exercise capacity, functionality, and general condition to the same extent in very old patients (*n* = 419 with age > 80 years), as in the younger patients with COPD. This is especially true if the percentage enhancement was regarded.

These results are not self-evident, since, due to the aging effects and numerous other negative physiological processes caused by lung diseases, an improvement was quite questionable. Despite the proven benefits of attending and adhering to rehabilitation by older patients, enrolment and adherence to PR in this demographic group remains suboptimal [27].

Prescribing behavior regarding PR is still very restrictive. In a large cohort study, it was shown that in the observation period from 2003 to 2012, there was only an increase from 1.02% to 2.03% of PR performed in patients over 85 years of age who had an indication for PR [28]. This is in line with older experiences in cardiology observing a bias against referring older patients for rehabilitation. In a review of all patients participating in a cardiac rehabilitation program, implicit selection against older patients was evident. Patients who were 70 years of age or older were 58% less likely to undergo a cardiac rehabilitation program regardless of important comorbid conditions [29]. Comparison of these two studies shows that there has been no significant improvement over a period of more than 15 years. This makes it more important to demonstrate again and again how effective PR can be in very old people.

In our study, with a similar intervention in all patients, at discharge, the very old patients’ group had lower walking distances measured for 6MWT, but similar degrees of relative improvement. However, the MID for 6MWT was exceeded, even in this group. The fact that the 6MWT was enhanced after the program demonstrates an improved ability to complete external work, is evidence of a training effect of PR, and indirectly represents a future with improved coping with the activities of daily living. Our findings are in line with the results found by Baltzan MA et al. showing a per cent improvement in 6MWT by the older patients (>80 years) of 40% (95% CI: –29% to 108%), which was comparable to the younger patients who improved by 60% (95% CI: 13% to 108%; *p* = 0.17), on average [30].

It is well known that both age and pulmonary diseases (e.g., COPD) have a negative impact on postural control, leading to an increased tendency to fall. Several studies demonstrated improvement in clinical balance measures after balance-specific training associated with PR in patients with COPD [31,32,33]. An increase in capacities and sensomotor strategies, safety regarding postural stability, physical demand, and respiratory function are needed [34]. Although our PR program did not include specific postural control training, the elderly patients in the >85-year-old group improved the most in FIM score with almost 9%. Since it is known that the evaluation of several risk factors for falls, including four FIM items: toileting, bed transfer, tub/shower transfer, and stairs, seems to have sufficient specificity and sensitivity by correctly predicting nearly 90% of patient falls [35], the results of our study suggest that the improvement achieved in the FIM score may also have a positive effect on the risk of falls in the elderly patients. A recent semi-structured interview-study in people with COPD and frailty found that this group was motivated to complete pulmonary rehabilitation but often require additional support and flexibility due to fluctuating and unpredictable health [36]. This is also our experience in caring for older patients with frailty. Person-centered approaches and regular readjustment of the PR contents according to the needs of the patients appear to be necessary.

The FT works well as an evaluative instrument to assess HRQL [37]. PR is known to improve HRQL in patients with COPD [38]. As expected, the score of the FT also improved within the PR in our cohort. However, it is also evident that the gain for the very old patient group was lower in our study, but the differences between the age-groups were not significant. For the group of the elderly patients, regarding the gain in quality of life, the results seem to be somewhat more inconsistent. A recent review including eight RCT recruiting four hundred and fourteen elderly patients found that PR resulted in significantly improved exercise capacity and quality of life in elderly people compared with the control group [39], whereas another study found a decline in quality of life in their collective [40]. We suspect methodological reasons as the basis of the different results regarding quality of life. There are considerable differences between the various quality of life assessments. In our study, the FT was used alone, but no single approach to interpretability is perfect. The use of multiple strategies is likely to enhance the interpretability of any instrument. Additionally, as a limitation of the Feeling Thermometer, difficulties for respondents in making precise distinctions between the measurement points to reflect their accurate feelings are described in comparison to sometimes more extensive questionnaires such as Chronic Respiratory Disease Questionnaire (CRQ) and St. George Respiratory Questionnaire (SGRQ) [41].

The duration of inpatient PR is an important predictor of outcome parameters as well. Although one can speculate that the possible gains from PR may not be apparent in older people until after a training period, the duration of PR was about the same in all groups. However, other studies have shown mixed results, regarding the necessity of a longer stay in hospitals or rehabilitation facilities. Compared with other studies, the duration of our PR program was similar [19,20,21,22].

There are some limitations to be discussed. First, this is a retrospective study so that some of the data were incompletely collected. However, only files that were complete concerning the assessments used were considered in this study (see the study flow chart in Figure 1). The second limitation is the long observation period of 7 years. It is not sure that all patients received the same PR content, as the PR program was developed and optimized over the years. Third, the single-center approach and the lack of a control group limit the validity of the data. The strength of the study is certainly the large number of patients in the older age group. Fourth, the 6MWT could be performed only once on admission to and once on discharge from PR. This might be a reason why the difference between the two tests overestimates the PR effects on the walking distance. However, for organizational reasons, only one test could be performed at a time in routine clinical practice.

## 5. Conclusions

This study shows that participation in a PR led to significant improvements according to the assessments regardless of the age-group in patients with COPD. Even in the age group of the very old patients (>80 years), significant enhancements were found.

## Figures and Tables

**Table 1:** Baseline characteristics on admission to PR.

*n*	3173
Diagnosis: ICD-10 J44.00–J44.99	3173
Age (mean (SD))	69 (12)
Age, *n* (%)	
≤60	679 (21)
61–70	908 (29)
71–80	1167 (37)
81–85	299 (9)
>85	120 (4)
Female (%)	1469 (46)
BMI (mean (SD))	25.22 (7)
BMI, *n* (%)	
<18.5	411 (13)
18.5–24.9	1309 (42)
25–29.9	771 (25)
30–34.9	374 (12)
35–39.9	145 (5)
40+	104 (3)
Length of Stay, days, (mean (SD))	18.85 (8)
Length of Stay, days, *n* (%)	
<14	706 (23)
<21	1747 (57)
<28	454 (15)
≥28	163 (5)

**Table 2:** Pre–post comparison results of FIM, 6MWT and FT in the age groups (provided in % and ∆FIM, ∆6MWT, and ∆FT).

	≤60 Years	61–70 Years	71–80 Years	81–85 Years	>85 Years	*p*
Total number (*n*)	679	908	1167	299	120	
FIM increase, % (SD)	4.94 [0.90, 11.24]	4.44 [0.00, 10.53]	4.55 [0.82, 11.40]	3.88 [0.00, 8.68]	7.41 [3.90, 15.36]	0.106
6MWT increase, % (SD)	36.10 [9.86, 79.71]	40.91 [16.13, 83.87]	43.14 [11.11, 95.29]	44.23 [15.86, 112.50]	57.89 [14.13, 125.66]	**0.040**
FT increase, % (SD)	24.04 [0.00, 50.00]	27.27 [0.00, 60.00]	28.57 [8.33, 63.07]	23.08 [0.00, 60.00]	20.00 [0.00, 57.78]	**0.021**
∆FIM (SD)	6 (14)	5 (13)	4 (18)	6 (17)	8 (7)	0.4
∆6MWT (SD)	106 (134)	110 (119)	98 (112)	104 (106)	92 (101)	0.2
∆FT (SD)	9 (28)	11 (27)	13 (27)	9 (28)	8 (32)	0.064

Legend: *n*, number; FIM, Functional Independent Measurement; 6MWT, Six-Minute Walk Test; FT, Feeling Thermometer; Statistical significance within the groups is marked by bold values.

## Data Availability

Supporting the reported results can be accessed by corresponding with the authors.
